# Comprehensive health assessment and blood analyte reference intervals of gopher tortoises (*Gopherus polyphemus*) in southeastern FL, USA

**DOI:** 10.1093/conphys/coab015

**Published:** 2021-03-26

**Authors:** Annie Page-Karjian, Kathleen Rafferty, Clerson Xavier, Nicole I Stacy, Jon A Moore, Sarah E Hirsch, Samantha Clark, Charles A Manire, Justin R Perrault

**Affiliations:** 1 Harbor Branch Oceanographic Institute, Florida Atlantic University, Fort Pierce, FL 34946, USA; 2 University of Florida College of Veterinary Medicine, Gainesville, FL 32609, USA; 3 Wilkes Honors College, Florida Atlantic University, Jupiter, FL 33458, USA; 4 Loggerhead Marinelife Center, Juno Beach, FL 33408, USA

**Keywords:** *Anaplasma*, epidemiology, *Herpesvirus*, *Mycoplasma*, *Ranavirus*, upper respiratory tract infection

## Abstract

The gopher tortoise (*Gopherus polyphemus*), a keystone species, is declining throughout its geographic range. Lack of knowledge with respect to the potential infectious diseases present within wild populations creates a dilemma for wildlife biologists, conservationists and public policy makers. The objective of this study was to conduct a health assessment of two previously unstudied gopher tortoise aggregations located at two sites in southeastern FL. Samples were collected from 91 tortoises (48 adults, 35 juveniles, 8 hatchlings) captured at Florida Atlantic University’s Harbor Branch Oceanographic Institute, in Fort Pierce, FL, USA in 2019, and Loggerhead Park in Juno Beach, FL, USA, during 2018–2019. Samples of blood, nasal swabs and oral/cloacal swabs were analyzed for hematology, plasma protein electrophoretic profiles and infectious disease testing including *Mycoplasma* spp. serology and polymerase chain reaction (PCR) assays for *Ranavirus*, *Herpesvirus* and *Anaplasma* spp. Hematological and plasma protein electrophoresis reference intervals are presented for adult and juvenile tortoises from both sites combined. Clinical signs consistent with upper respiratory tract disease (URTD) were observed in 18/91 (20%) tortoises, and antibodies to *Mycoplasma agassizii* were detected in 33/77 (42.9%) tortoises. Adult tortoises were significantly more likely than juveniles to have URTD clinical signs, and statistically significant, positive relationships were observed between the presence of antibodies to *Mycoplasma* spp. and carapace length, packed cell volume and plasma globulin concentrations. *Anaplasma* spp. inclusions were observed in 8/82 (10%) tortoises, but PCR detected *Anaplasma* sp. in 21/83 (25%) tortoises. *Herpesvirus* and *Ranavirus* were not detected in any blood or swab samples. This work contributes important baseline information on the health of gopher tortoises toward the southern end of the species’ range.

## Teaser

Wildlife population health assessment is critical for understanding threats to their survival. A health assessment was conducted on two gopher tortoise (*Gopherus polyphemus*) aggregations in southeastern FL. Blood analyte reference intervals and pathogen prevalence data provide baseline health information and insight on gopher tortoise health in the species’ southern geographic range.

## Introduction

The gopher tortoise (*Gopherus polyphemus*) is declining throughout its range due to habitat loss and fragmentation, human interaction including vehicular collision, predation by domestic animals and disease (Auffenberg and Franz, 1982; Diemer-Barish *et al*., 2010; Smith *et al*., 2006). Gopher tortoises are federally listed in the western portion of their range, state-listed in FL and currently a candidate for federal listing in the eastern portion of their range (U.S. Fish & Wildlife Service, 2011). The inherent impacts of infectious diseases on wildlife conservation and biodiversity are evident; however, until recently, these impacts were not often considered. Lack of knowledge with respect to the potential infectious diseases present within wild populations, the impact of disease status on relocation or reproduction of species and disease impacts to long-term population viability create a major dilemma for wildlife biologists, conservationists and public policy makers. This is especially critical for a keystone species such as the gopher tortoise (Eisenberg, 1983).

Most disease researches in wild gopher tortoises have focused on upper respiratory tract disease (URTD) caused by *Mycoplasma agassizii* and *Mycoplasma testudineum* (McLaughlin, 1997; Smith *et al*., 1998; Brown *et al*., 1999; Berish *et al*., 2000; McLaughlin *et al*., 2000; Wendland, 2007). These contagious bacteria, transmitted via direct contact between tortoises (McLaughlin, 1997), can infect the respiratory tract of tortoises (Brown *et al*., 2001) and cause mild to severe nasal and ocular discharge, conjunctivitis and swelling of the eyes and nares (Jacobson *et al*., 1991; Schumacher *et al*., 1997; McGuire *et al*., 2014a). Mycoplasmosis is perhaps the most important chronic infectious disease of wild and captive tortoises in North America and Europe (Jacobson *et al*., 2014). Diagnostic tests specifically validated for gopher tortoise URTD are available, including polymerase chain reaction (PCR) to detect *Mycoplasma* spp. DNA in nasal sections (Brown *et al*., 1995) and an enzyme-linked immunosorbent assay (ELISA) that detects antibodies to *M. agassizii* 6–8 weeks post-exposure (Schumacher *et al*., 1993; Wendland *et* al., 2007). Although it is considered a primary cause of disease, serological tests have indicated that exposure to *Mycoplasma* spp. is widely distributed within the gopher tortoise’s range and exposed animals are not always clinically ill (Jacobson *et al*., 2014). Exposed (i.e. antibody-positive) gopher tortoises have been found in MS (Smith *et al*., 1998), GA (McGuire *et al*., 2014a, 2014b), AL (Goessling *et al*., 2019) and throughout much of FL (Beyer, 1993; Epperson, 1997; Smith *et al*., 1998; Berish *et al*., 2000; McCoy *et al*., 2007; Wendland 2007). Previous investigations have demonstrated antibody prevalence of 30% and 22% in FL tortoises (Berish *et al*., 2000; Wendland, 2007), and a more recent study demonstrated URTD prevalence in FL gopher tortoises ranged from 0% to 78%, depending on site (Diemer-Barish *et al*., 2010).

A number of other pathogens are known to cause (e.g. *Herpesvirus*) or potentially cause (e.g. *Ranavirus, Helicobacter* sp.) similar clinical signs to URTD (Jacobson, 1994; Pettan-Brewer *et al*., 1996; Westhouse *et al*., 1996; Origgi and Jacobson, 2000; Origgi *et al*., 2004; Johnson, 2006; Wellehan *et al*., 2016). For example, *Ranavirus* has been associated with nasal and ocular discharge, conjunctivitis and subcutaneous edema in tortoise species, including gopher tortoises (Westhouse *et al*., 1996; Johnson *et al*., 2008). In several other species of tortoise, *Herpesvirus* infections can result in necrotizing stomatitis, glossitis, tracheitis, laryngitis and rhinitis (Jacobson *et al*., 1985, Drury *et al*., 1998, Muro *et al*., 1998, Johnson *et al*., 2005). However, because diagnostic tests are not readily available, little is known about the importance and prevalence of these microorganisms in wild tortoises. Other pathogens reported in gopher tortoises include intestinal parasites such as pinworms, ascarids, flukes and protozoans such as *Cryptosporidium* spp., a zoonotic pathogen (McGuire *et al*., 2013, Huffman, 2017); various hemoparasites including *Anaplasma* spp., which has been associated with anemia, and hemogregarines, which are typically considered an incidental finding (Cooney *et al*., 2016, 2019, Raskin *et al*., 2020), and ectoparasites including ticks (e.g. *Amblyomma tuberculatum*; Ennen and Qualls, 2011). In cases of an immunocompromised and/or stressed host, nutritional imbalance, reduced body condition and/or presence of co-infections, these pathogens may cause chronic disease, which can lead to reduced reproductive capacity, abnormal growth and development, increased susceptibility to secondary infections and, in some cases, a reduced life span (U.S. Fish & Wildlife Service, 2019). Identifying the impacts of such diseases can be a difficult task. The full effect of chronic disease on a long-lived species such as the gopher tortoise may take months to years to manifest in a population. Therefore, it is important that populations are monitored using standardized techniques so that any changes associated with health problems may be detected over time (Wendland *et al*., 2009).

Despite seemingly healthy adult tortoise populations in many areas of south FL, low fecundity has been observed in several fragmented habitats, demonstrated by a lack of nests within active burrows and a lack of juveniles and sub-adults (Zeiger and Frazier, 2012). This top-heavy demographic structure raises concern about population sustainability. Assessment of fecund tortoise populations living in fragmented habitats will generate data to help explain health and fecundity differences observed in geographically and ecologically similar sites. The objective of this study was to conduct a comprehensive health assessment of two previously unstudied gopher tortoise aggregations in southeastern FL.

## Materials and methods

### Study sites

**Figure 1:**
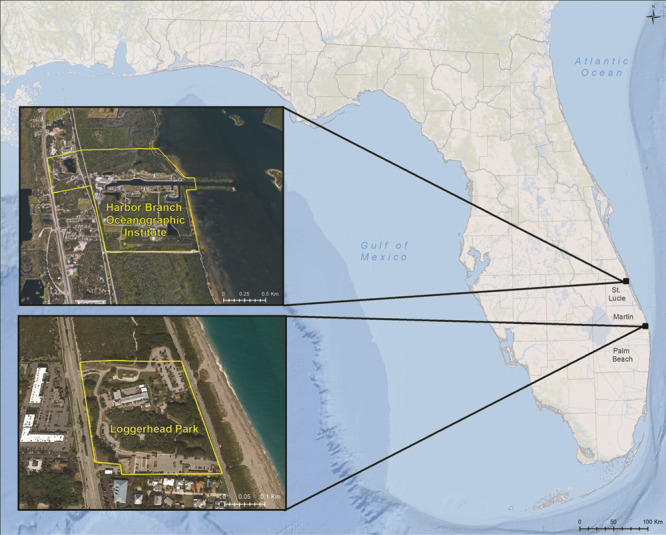
Map depicting sampling sites of two gopher tortoise aggregations in southeastern FL, USA.

To obtain estimated population sizes, we conducted surveys over the 17.3-acre area of Loggerhead Park, Juno Beach, FL (26.8847°N, −80.0563°W), during March 2018–August 2019, and over the 144-acre campus of Florida Atlantic University’s Harbor Branch Oceanographic Institute (HBOI; 27.5360°N, −80.3614°W) (Fig. 1) during May–August 2019. The 17.3-acre Loggerhead Park consists of ~6.1 acres of marginally to moderately suitable habitat including sandy pine-scrub, oak maritime forest and sandhills (Ashton *et al*., 2008). This area is bounded by highly developed commercial parking lot areas on the north and south sides; by a 4-lane highway with very heavy traffic on the western side, across from which lie commercially developed paved sites; and by a 2-lane road on the eastern side, across from which is a sandy beach and the Atlantic Ocean (Fig. 1). Loggerhead Park is also surrounded by dense patches of saw palmetto plants (*Serenoa repens*), which effectively block gopher tortoise movement in many areas of the park. The 144-acre HBOI site offers ~122.1 acres of contiguous, moderately suitable gopher tortoise habitat consisting of sandy live oak (*Quercus* spp.) hammock mixed with stands of longleaf (*Pinus palustris*) and loblolly (*Pinus taeda*) pines and shrubby mesic rangeland. On the western boundary of the HBOI campus, there is a 4-lane highway with relatively light traffic, on the other side of which are two wildlife refuges (Indrio Savannahs Preserve and Lake Indrio Preserve), which both also host gopher tortoise aggregations (J. Moore, personal observation).

### Tortoise capture and physical examination

Tortoises were hand-captured opportunistically as they were visually encountered. All live tortoises encountered at the surface or in burrow entrances within the geographic and temporal bounds of the study were enrolled in the health assessment. Tortoises at Loggerhead Park were sampled throughout the year, while tortoises at HBOI were only sampled during summer months. Upon capture, animals were transported to a shaded area for physical examination and sample collection. Prior to and after health assessment, each tortoise was held in a clean plastic bin (dimensions: 2 × 2 × 1.5 ft), which was disinfected between every tortoise using a 1:20 dilution of 5% household bleach in water. Complete physical examinations were conducted on all tortoises following the guidelines provided by Wendland *et al.* (2009), including observation of overall posture, behavior, ambulatory ability and breathing sounds and closer observation for any clinical signs suggestive of URTD (e.g. nasal discharge, conjunctivitis, swollen eyes, lethargy, labored/wheezy breathing) and lesions suggestive of chronic URTD (e.g. nasal scarring and asymmetric nares). Body measurements were taken, including body mass (to the nearest 0.1 kg) using a digital scale, straight carapace length (SCL, to the nearest 1 mm) and plastron length (to the nearest 1 mm) (McRae *et al*., 1981). Sex was determined in adult tortoises based on external morphology such as gular length, plastron concavity and visual observation of reproductive organs in males (McRae *et al*., 1981; Eubanks *et al*., 2003). All *Amblyomma tuberculatum* ticks were recorded and collected (Keirans and Litwak, 1989). Age class was determined based on carapace length, with tortoises equal to and greater than 235 mm SCL considered sexually mature adults (Moore *et al*., 2009). All captured adult tortoises were permanently and uniquely marked using a triangular file or Dremel tool to notch one or a combination of the eight rearmost marginal scutes, following the Florida Fish and Wildlife Commission (FWC)’s gopher tortoise marking guidelines (FWC, 2008). Prior to notching, the shell was swabbed with povidone iodine and care was taken to avoid injury to the limbs.

### Sample collection

Venous blood samples (0.5–4 mL; <1% of body weight) were collected from the brachial (*N* = 46) or jugular (*N* = 41) vein using a 25- or 22-gauge, 1-inch needle fitted to a 6-ml syringe and aseptic technique (Wendland, 2007; Wendland *et al*., 2009). Blood samples were collected within 5–10 minutes after tortoises were captured, placed into lithium heparinized tubes, wrapped in bubble wrap, stored on ice packs and transported to the laboratory. From lithium heparin tubes, ~300 μL of blood were aliquoted into sterile cryovials and stored in an ultralow freezer (−80°C) for up to 20 months prior to DNA extraction and molecular analysis (detailed below). Within <1 hour after sample collection, a small amount of whole blood from the lithium heparin tubes was placed into two capillary tubes and two blood films were prepared. To determine packed cell volume (PCV, %), whole blood samples in capillary tubes were centrifuged for 5 minutes at 1300 *g* (5,000 rpm) in a microhematocrit centrifuge and interpreted using a hematocrit microcapillary tube reader. After centrifugation, plasma color was assessed visually for signs of hemolysis, which can influence blood chemistry and protein data (Stacy et al. 2019). Plasma total protein concentration (TP-R) was determined by refractometer (Loggerhead Park, Reichert VET 360; HBOI, Brix Clinical Refractometer). Within two hours of blood collection, the remaining blood samples were centrifuged for 5 minutes (Loggerhead Park, LW Scientific C5 centrifuge at 4200 *g* [5000 rpm]; HBOI, The Drucker Co. Horizon 642VES at 4200 *g* [5000 rpm]). Separated plasma was immediately removed from spun tubes, plasma color was recorded again and 200–500 μL aliquots were placed into cryovials and frozen in an ultralow freezer (−80°C) for 6–22 months prior to further analysis. Nasal swabs were collected from all tortoises by swabbing the external nares and the anterior-most portion (anterior 3 mm) of the internal nares, using sterile thin cotton-tipped applicator swabs (Puritan™ 25 826 5WC, Guilford, ME, USA). A single sterile cotton-tipped applicator (Puritan™ 25 806 10WC, Guilford, ME, USA) was used to swab both the oral cavity and the cloaca, consecutively, using gentle pressure. After collection, swabs were placed into cryovials and frozen in an ultralow freezer for up to 12 months prior to further analysis. Any ectoparasites observed during physical examination were removed using forceps and placed into separate glass specimen jars containing 70% ethyl alcohol. After sample collection, tortoises were hydrated in warm water for 15–20 minutes then released at the site of capture (Wendland *et al*., 2009).

### Hematology and plasma protein electrophoresis

Blood films were stained using Wright–Giemsa stain (Harleco®, EMD Millipore, Billerica, MA, USA). Light microscopy was used to conduct complete blood cell counts and evaluation of any hemoparasites by one evaluator. Evaluation of blood films included white blood cell (WBC) estimate (Weiss, 1984) and differential (including mature heterophils, immature heterophils, lymphocytes, monocytes, eosinophils and basophils) based on 200 WBC counts, and morphological evaluation of red blood cells (RBCs), WBCs and thrombocytes. Immature heterophils were quantified as a separate WBC category in addition to mature heterophils (Stacy *et al*., 2017), and immature RBCs were counted as number of immature RBC per 100 mature RBCs. The heterophil:lymphocyte ratio was calculated using numbers of mature and immature heterophils combined.

Hemoglobin concentration was analyzed in ~20 μL of previously frozen, thawed whole blood using a HemoCue® Hb 201^+^photometer (HemoCue®, Inc., Lake Forest, CA, USA) with HemoCue® Hb 201 microcuvettes, which has been validated for use in birds and used in sea turtles (Velguth *et al*., 2010, Harter *et al*., 2015, Stacy *et al*., 2019, Page-Karjian *et al*., 2020) and has a measuring range of 0–256 g l^−1^. Frozen plasma aliquots (0.5–1.0 mL) were shipped overnight on dry ice to the University of Miami Avian & Wildlife Laboratory (UMAW), where they were analyzed for protein fractions using the SPIFE 3000 system (Helena Laboratories Inc., Beaumont, TX, USA) and accompanying gels (Dickey *et al*., 2014), with protein fraction delimits placed using the following conventions: pre-albumin, albumin and alpha-1, alpha-2, beta and gamma globulins; total globulins were calculated. Total protein was quantified using the Biuret method (TP-B) at UMAW, and the albumin:globulin (A:G) ratio was calculated for each sample.

### Parasite and pathogen analyses

Frozen plasma aliquots were shipped overnight on dry ice to the University of Florida College of Veterinary Medicine, Mycoplasma Research Laboratory where they were analyzed for antibodies to *Mycoplasma agassizii* and *M. testudineum* using ELISA testing. The sample results of the ELISAs were expressed as ratios between the absorbance value of the test sample and that of a negative control (Schumacher *et al*., 1993); results based on antibody tests were grouped into one of three classes based on antibody titers: positive (>32), negative (<32) and suspect (=32) (Wendland *et al*., 2007; McGuire *et al*., 2014a).

Genomic DNA (gDNA) was extracted from the whole blood samples using the DNEasy Blood and Tissue Kit (Qiagen) and from the oral/cloacal and nasal swabs using the MinElute Virus Spin Kit (Qiagen), following manufacturer’s instructions. Resultant gDNA concentration was measured in each sample using absorbance spectrophotometry (Nanodrop) and ratios of absorption at 260 nm versus 280 nm were evaluated to ensure DNA purity. Extracted gDNA samples were stored at −80°C for up to 3 months prior to qPCR analysis. Thawed gDNA samples were analyzed for *Ranavirus* DNA using a quantitative polymerase chain reaction (qPCR) assay, after Allender *et al*. (2013). Specifically, a primer/probe-based qPCR assay (TaqMan® primers, FAM dye labeled probe, Integrated DNA Technologies™) was applied to gDNA extracted from whole blood and oral/cloacal and nasal swab samples. This assay targets a 70-bp segment of the major capsid protein of frog virus 3 (GenBank accession numbers AY150217.1 and NC_005946.1). Samples were tested in triplicate on an AriaMX Real-Time PCR System (Agilent Technologies) using 11.8 μL of Lo-ROX Probe qPCR master mix (Bioline), 0.8 μL each of forward and reverse primers, 0.2 μL of probe and 8.2 μL of template per reaction, and the following thermal cycling conditions: 1 cycle at 50°C for 2 min followed by 40 cycles each of 95°C for 15 s and 60°C for 60 s. Nuclease-free water was used as a no-template control, and a 1:10 serial dilution of a 70 bp synthetic preparation of the target gene segment (gBlock; Integrated DNA Technologies™) was used as a positive control and to construct an intra-assay standard curve that was applied to each run.

Genomic DNA samples extracted from oral/cloacal swabs were shipped overnight on dry ice to the University of Georgia College of Veterinary Medicine, Infectious Diseases Laboratory in Athens, GA, USA. There, the samples were analyzed using a generic nested PCR based on conserved amino acid sequences from the *Herpesvirus* DNA polymerase gene (Van Devanter *et al*., 1992), which will theoretically detect any *Herpesvirus*. Genomic DNA samples extracted from whole blood samples were shipped overnight on dry ice to the Zoological Medicine Laboratory at the University of Florida College of Veterinary Medicine in Gainesville, FL, USA. There, the samples were analyzed using quantitative TaqMan PCR assays targeting the tortoise *Anaplasma* groEL and sucB genes (Crosby *et al*., 2016).

### Statistical analyses

Measures of central tendency and range were calculated for SCL (mm) and body mass (kg) in juvenile and adult tortoises from each sampling site. Parametric methods for sample sizes ≥20 but <120 were used to calculate blood analyte reference intervals for juveniles and adults from both sites combined (Friedrichs *et al*., 2012). Normality was assessed using the Shapiro–Wilk test, while outliers were detected using the Dixon–Reed test and subsequently removed from calculation of reference intervals. Mean and standard deviation were calculated for hematological data for hatchlings sampled in Loggerhead Park. Spearman rank-order correlations were employed to determine relationships between SCL and the blood analytes. Power regression was used to analyze the relationship between SCL and mass, while linear regression was used to analyze the relationships between PCV and hemoglobin concentration and TP-R and TP-B for the two sites. This was done because, due to geographic separation and temporal overlap of sampling efforts, refractometer brands available at the two sites differed. To evaluate whether blood collection site (jugular vein versus brachial vein) influenced clinical pathology data, we compared clinical pathology parameters between tortoises sampled from the two anatomic sites, separated by age class, using Mann–Whitney *U*-tests.

Fisher’s exact tests were used to compare age class proportions between the two sampling sites, and to evaluate for associations between the results of *Mycoplasma* spp. serology tests and tortoise sex and presence/absence of URTD clinical signs. Additionally, logistic regression analyses were used to test the relationships between *Mycoplasma* spp. serology test results (e.g. positive, negative) and SCL, PCV, estimated tWBC, absolute heterophil and lymphocyte counts and plasma total globulin concentrations. A Fisher’s exact test was also used to evaluate for a statistical association between the presence/absence of ticks, and the presence/absence of intraerythrocytic hemogregarine gametocytes noted on examination of blood films. Logistic regression was used to evaluate the relationship between PCV data and the results of *Anaplasma* spp. diagnostic tools (presence of intraerythrocytic inclusions, qPCR). Cohen’s Kappa coefficient was calculated to analyze the level of diagnostic agreement between microscopic evaluation of blood films and qPCR for *Anaplasma* spp. Fisher’s exact tests were then used to evaluate the relationships between *Anaplasma* spp. infection (diagnosed via either microscopic evaluation or qPCR) and presence/absence of ticks and between *Anaplasma* spp. infection and *Mycoplasma* spp. serology results. For all statistical tests, alpha was set at 0.05.

### Ethics statement

Sample and data collection and use were conducted by authorized personnel under a Scientific Collection permit (#LSSC-17-00046A) issued by Florida Fish & Wildlife Conservation Commission and approved by the Florida Atlantic University Institutional Animal Care and Use Committee under protocol #A17–11.

## Results

Overall, 91 tortoises were captured and evaluated for this study, including 57 at Loggerhead Park and 34 at HBOI, representing three age classes (Table 1). The HBOI campus had significantly fewer juvenile tortoises (including hatchlings) and more adults—nearly 3 adults for every juvenile compared to Loggerhead Park (approximately 1.2 juveniles for every adult) (*P* = 0.003). Physical examination revealed that 18/91 (19.8%) of the tortoises had clinical signs consistent with URTD, including nasal discharge (*N* = 10), asymmetrical nares (*N* = 6), wheezing (*N* = 5), palpebral/conjunctival swelling (*N* = 3) and ocular discharge (*N* = 2). Additionally, 12/91 (13.2%) tortoises had some other form of physical abnormality noted during physical examination, including limb (*N* = 1), eye (*N* = 1) and shell abnormalities (*N* = 3), or extra scutes (*N* = 7). The five tortoises sampled at Loggerhead Park that tested ‘suspect’ for antibodies to *M. testudineum* were also positive for antibodies to *M. agassizii*; however, none of these five tortoises exhibited clinical signs of URTD at the time of sampling. All sampled ticks were identified as gopher tortoise ticks (*A. tuberculatum*).

**Table 1:** Descriptive statistics resulting from physical examination and pathogen surveys of two gopher tortoise (*G. polyphemus*) aggregations in southeastern FL, USA

	Loggerhead Park *N* = 57	HBOI Campus *N* = 34
Physical examination		
Age class		
Adults	23 (40%)^*^	25 (73.5%)^*^
Juveniles	28 (49%)^*^	7 (20.6%)^*^
Hatchlings	6 (11%)^*^	2 (5.9%)^*^
Sex		
Males	15 (26%)	8 (24%)
Females	10 (18%)	17 (50%)
Unknown	32 (56%)	9 (26%)
Pathogen survey		
*A. tuberculanum* ticks	1 (2%)^*^	20 (59%)^*^
Intraerythrocytic hemogregarine gametocytes	0/49 (0%)^*^	10/33 (30%)^*^
*M. agassizii* ELISA ‘positive’ (titers > 32)	24/47 (51%)	19/30 (63%)
*M. agassizii* ELISA ‘suspect’ (titers = 32)	6/47 (13%)	5/30 (17%)
*M. testudineum* ELISA ‘positive’ (titers > 32)	0/47 (0%)	0/30 (0%)
*M. testudineum* ELISA ‘suspect’ (titers = 32)	5/47 (11%)	0/30 (0%)
*Ranavirus* qPCR	0/53 (0%)	0/29 (0%)
*Herpesvirus* cPCR	0/53 (0%)	0/29 (0%)
Intraerythrocytic inclusions suggestive of *Anaplasma* spp.	7/49 (14%)	1/33 (3%)
*Anaplasma* spp. qPCR	12/53 (23%)	9/30 (30%)

^*^Statistically significant differences between sampling sites.

**Figure 2:**
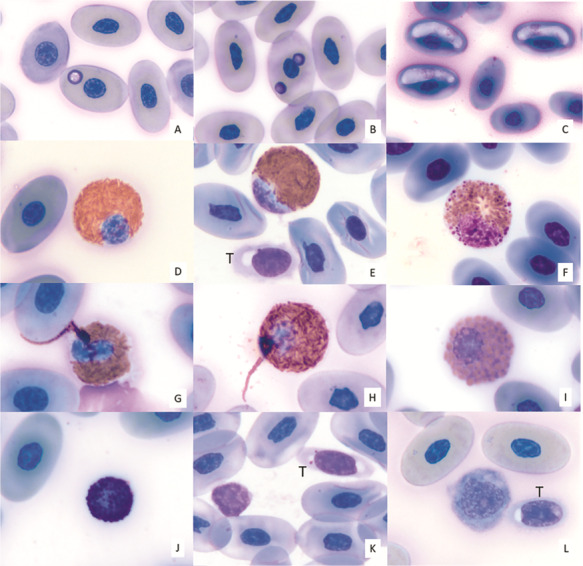
Composite of photomicrographs of blood films from gopher tortoises (*G. polyphemus*) in this study. (**A**) Erythrocyte with granular inclusion most consistent with *Anaplasma* spp. (confirmed by PCR); (**B**) Erythrocyte with two granular inclusions most consistent with *Anaplasma* spp. (confirmed by PCR); (**C**) Erythrocytes with hemogregarine gametocytes; (**D**) Mature heterophil; (**E**) Immature heterophil and thrombocyte (T); (**F**) Immature heterophil with primary granules; (**G**, **H**) Heterophils with ‘whip-like’ projections; (**I**) Eosinophil; (**J**) Basophil; (**K**) Small lymphocyte and thrombocyte (T); (**L**) Monocyte and thrombocyte (T). ×100 objective. Wright–Giemsa stain.

**Figure 3:**
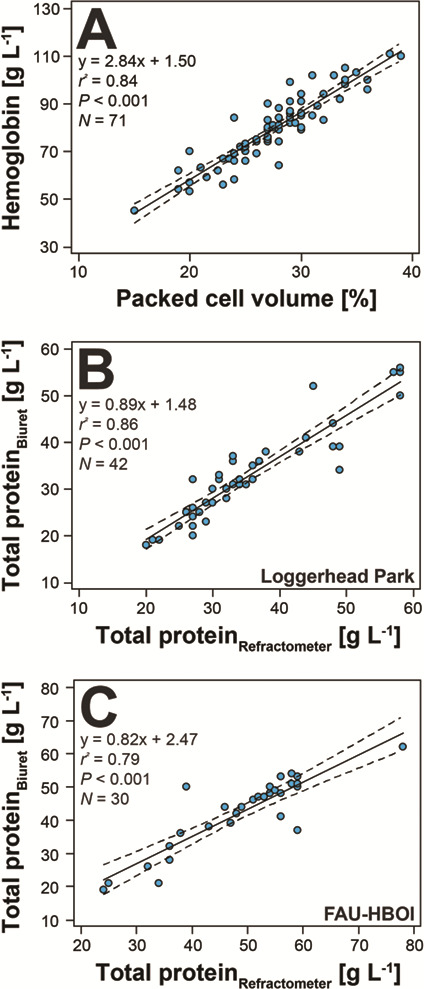
Linear regression analysis revealed strong positive relationships between (**A**) PCV and plasma hemoglobin concentration and between plasma total protein by refractometer and total protein by biuret method for both (**B**) Loggerhead Park and (**C**) HBOI gopher tortoises (*G. polyphemus*). Total protein relationships by refractometer were analyzed separately for the two aggregations as different brands of refractometer were used.

All plasma samples had hemolysis scores of either 0 or 1+, and no lipemia was documented. Mann–Whitney *U*-tests revealed significant differences between the jugular and brachial vein blood sampling sites in adult tortoises [samples from brachial veins had higher median immature heterophils (*U* = 127.5, *N* = 44, *P =* 0.026), gamma globulins (*U* = 84.5, *N* = 44, *P =* 0.001) and total globulins (*U* = 137.5, *N* = 44, *P =* 0.049) and lower median A:G ratio (*U* = 130.5, *N* = 44, *P =* 0.032) and pre-albumin (*U* = 126.5, *N* = 44, *P =* 0.025) than samples from jugular veins] and juveniles [samples from brachial veins had lower median heterophils (*U* = 46.5, *N* = 32, *P =* 0.033), alpha-1 globulins (*U* = 36.5, *N* = 29, *P =* 0.041) and beta globulins (*U* = 34.5, *N* = 29, *P =* 0.032)]. These differences, however, did not consistently indicate that either sampling site influenced clinical pathology results via lymph dilution. Two samples suspected to have slight lymph dilution based on blood color during sampling also had low PCVs (10% and 12%) and were not included in hematological and plasma biochemical analyses. Hematological reference intervals for juveniles and adults are presented in Table 2. There were four juvenile tortoises, two at HBOI and two at Loggerhead Park, that had PCV values less than 20%, ranging from 15%–19%. None of these samples were suspected of lymph dilution, which can alter blood analyte values (Gottdenker and Jacobson, 1995). Of those four tortoises, none had clinical signs or URTD, none had hemoparasites or intraerythrocytic inclusions observed on blood films and all tested negative for antibodies to *Mycoplasma* spp. and for *Anaplasma* spp. via qPCR. Polychromasia (erythrocyte color variation) was absent (*N* = 1), minimal (*N* = 2) or mild (*N* = 1); and anisocytosis (erythrocytes unequal in size) was absent (*N* = 1), minimal (*N* = 1) or mild (*N* = 2). One of the tortoises had two ticks removed at physical examination, while the other three had no ticks observed. Results of morphological evaluation of RBCs, WBCs and thrombocytes are shown in Table 3. Figure 2 depicts erythrocytes containing inclusions suggestive of *Anaplasma* spp. (A, B) as well as hemogregarine gametocytes (C), and Fig. 2D–J shows various examples of WBCs observed in gopher tortoises in this study. Linear regression analysis revealed very strong positive relationships between PCV and plasma hemoglobin concentration (Fig. 3a) and between TP-R and TP-B for Loggerhead Park tortoises (Fig. 3b) and HBOI tortoises (Fig. 3c). Additionally, the hemoglobin concentration is about three times the PCV (PCV * 2.9) using the SI unit (g l^−1^), or a third of the PCV (PCV * 0.29) using the conventional unit (g dl^−1^).

**Table 2:** Reference intervals with 90% confidence interval for upper and lower limits for PCV (*N* = 35 for juveniles; *N* = 48 for adults), hemoglobin (*N* = 26 for juveniles; *N* = 44 for adults), WBC count with differentials (*N* = 36 for juveniles; *N* = 44 for adults) and plasma protein electrophoresis (*N* = 27 for juveniles; *N* = 44 for adults) in standard international units for juvenile and adult gopher tortoises (*G. polyphemus*) from southeastern FL, USA. Mean hematological data are provided for six hatchling gopher tortoises

	Juveniles			Adults			Hatchlings
	RI	LRL 90% CI	URL 90% CI	RI	LRL 90% CI	URL 90% CI	Mean ± SD
Hematology							
PCV (%)	16–34	14–19	32–36	21–37	19–23	36–39	20 ± 9
Hemoglobin (g L^−1^)	46–99	38–53	91–107	59–110	53–64	105–116	11 ± 9
tWBC (x10^3^ μ1^−1^)	4.31–17.31	2.72–5.90	15.72–18.90	5.07–21.13	4.33–5.94	18.05–24.73	9.85 ± 5.17
Total heterophils (×10^3^ μ1^−1^)	0.78–6.59	0–1.56	5.81–7.37	1.39–8.88^a^	1.13–1.70	7.23–10.90	3.22 ± 2.48
Lymphocytes (×10^3^ μ1^−1^)	1.61–8.26^a^	1.32–1.97	6.76–10.09	1.24–6.57	0.65–1.83	5.99–7.16	3.30 ± 2.20
Monocytes (×10^3^ μ1^−1^)	0–1.22	0–0.15	1.07–1.36	0.12–1.86^a^	0.09–0.16	1.37–2.53	0.76 ± 0.39
Eosinophils (×10^3^ μ1^−1^)	0.21–4.66^a^	0.15–0.31	3.18–6.84	0.29–5.20^a^	0.21–0.40	3.76–7.18	0.90 ± 0.64
Basophils (×10^3^ μ1^−1^)	0.17–4.22^a^	0.12–0.26	2.86–6.24	0.19–3.36^a^	0.14–0.26	2.45–4.62	1.53 ± 1.14
Heterophil:lymphocyte	0.25–3.56^a^	0.17–0.35	2.49–5.10	0.32–2.88^a^	0.25–0.41	2.26–3.67	0.98 ± 0.89
Plasma proteins							
Total protein (g L^−1^)	15.8–37.0	12.8–18.8	34.0–40.1	27.2–60.2	23.6–30.9	56.6–63.9	--
Albumin:globulin	0.35–1.27	0.22–0.48	1.14–1.40	0.23–0.74^a^	0.20–0.26	0.65–0.84	--
Pre-albumin (g L^−1^)	2.9–9.7	1.9–3.8	8.8–10.7	4.1–9.5^a^	3.8–4.5	8.7–10.5	--
Albumin (g L^−1^)	2.9–7.3	2.3–3.6	6.6–7.9	3.6–9.9^a^	3.2–4.0	8.8–11.1	--
Alpha-1 globulins (g L^−1^)	0.5–2.3	0.3–0.8	2.1–2.6	0.7–2.4	0.6–0.9	2.2–2.5	--
Alpha-2 globulins (g L^−1^)	1.1–4.9	0.6–1.6	4.4–5.5	2.5–7.5	1.9–3.0	7.0–8.1	--
Beta globulins (g L^−1^)	2.6–12.5	1.2–4.0	11.1–13.9	8.1–29.6	5.7–10.4	27.2–31.9	--
Gamma globulins (g L^−1^)	1.1–4.8	0.5–1.6	4.3–5.3	2.6–8.5	1.9–3.2	7.8–9.1	--
Total globulins (g L^−1^)	7.5–22.3	5.4–9.6	20.2–24.3	16.2–46.0	12.9–19.4	42.7–49.3	--

a ^a^ Reference intervals were calculated using logarithmic transformations, as original data were non-normal.

Abbreviations: CI, confidence interval; LRL, lower reference limit; RI, reference interval; URL, upper reference limit; SD, standard deviation.

**Table 3:** Morphological evaluation of RBCs, WBCs and thrombocytes for two gopher tortoise (*G. polyphemus*) aggregations in southeastern FL, USA. For immature RBC/100 mature RBC and hemogregarines/100 RBC, mean ± standard deviation are reported, with the range parenthetically

	Loggerhead Park	HBOI Campus
Thrombocytes	Adequate: 100% (49/49)	Adequate: 100% (33/33)
Polychromasia^*^	Absent: 31% (15/49)^*^ Minimal: 33% (16/49) Mild: 29% (14/49) Moderate: 8% (4/49)	Absent: 9% (3/33)^*^ Minimal: 36% (12/33) Mild: 55% (18/33)
Anisocytosis^*^	Minimal: 55% (27/49)^*^ Mild: 37% (18/49)^*^ Moderate: 8% (4/49)	Absent: 6% (2/33) Minimal: 21% (7/33)^*^ Mild: 73% (24/33)^*^
Immature RBC/100 mature RBC	3.8 ± 5.7 (0–27)	2.8 ± 1.8 (0–7)
Erythrocyte morphology	NSCF: 96% (47/49) Rare early stage precursors: 2% (1/49) Occasional variably sized clear RBC vacuoles of unknown significance; one to multiple per RBC: 2% (1/49)	NSCF: 97% (32/33) Rare early stage precursors: 3% (1/33)
RBC inclusions suggestive of *Anaplasma* spp.	0: 86% (42/49) <1: 8% (4/49) 1–3: 4% (2/49) 3–5: 2% (1/49)	0: 97% (32/33) < 1: 3% (1/33)
Hemogregarine parasitemia^*^	Absent: 100% (49/49)^*^	Absent: 70% (23/33) Rare: 9% (3/33) Occasional: 9% (3/33) Few: 6% (2/33) Frequent: 6% (2/33)
Hemogregarine gametocytes/100 RBC^*^	0 (0)	2.3 ± 5.8 (0–24)
Heterophil projections^*^	Absent: 79% (38/48) Few: 21% (10/48)^*^	Absent: 100% (33/33)^*^
Other WBC morphological findings	NSCF: 100% (48/48)	NSCF: 100% (33/33)

Abbreviations: NSCF, no significant clinical findings.

^*^Statistically significant differences between sampling sites by specific categories (e.g. absent, minimal, mild, etc.).

**Figure 4:**
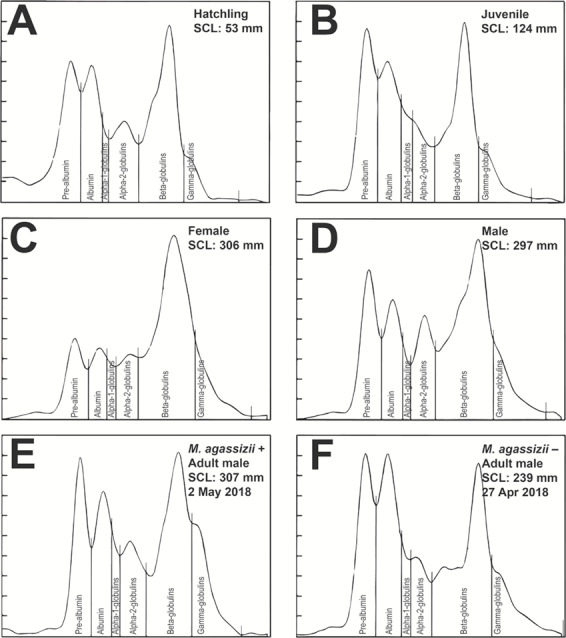
Representative plasma protein electrophoretograms of (**A**) hatchling, (**B**) juvenile, (**C**) adult female and (**D**) adult male gopher tortoises (*G. polyphemus*) from southeastern FL showing the fractions of interest: pre-albumin, albumin, alpha-1 globulins, alpha-2 globulins, beta globulins and gamma globulins. An adult male tortoise that was seropositive for *M. agassizii* (**E**) had notably larger fractions of alpha-2 globulin, beta globulin and gamma globulin compared (**F**) to an adult male tortoise that was seronegative for *M. agassizii*. All data were determined in non-hemolyzed plasma samples. By convention, no units are reported on the y-axis (Gicking *et al*., 2004).

PCV, immature heterophil counts, plasma concentrations of hemoglobin, total protein, albumin, alpha-2 globulin, beta globulin, gamma globulin, total globulins and A:G were significantly correlated to SCL, a proxy for age (Tuberville *et al*., 2011) (Table 4). Examples of protein electrophoretograms for hatchling, juvenile and adult gopher tortoises from this study are shown in Fig. 4, demonstrating the progression toward increased plasma proteins as animals mature, especially with regards to beta globulin and gamma globulin.

**Table 4:** Significant Spearman correlations between SCL (a proxy for age) and measured blood analytes for two gopher tortoise (*G. polyphemus*) aggregations in southeastern FL, USA. Blood values were combined for the two aggregations

Analyte	*r_s_*	*P*	*N*
PCV	0.38	<0.001	84
Hemoglobin	0.36	0.002	71
Total protein (refractometer) LMC	0.70	<0.001	51
Total protein (refractometer) HBOI	0.58	<0.001	32
Immature heterophils	0.32	0.003	81
Total protein (biuret)	0.75	<0.001	72
Albumin:globulin	−0.71	<0.001	72
Albumin	0.30	0.009	72
Alpha-2 globulins	0.70	<0.001	72
Beta globulins	0.74	<0.001	72
Gamma globulins	0.68	<0.001	72
Total globulins	0.77	<0.001	72

A significantly positive relationship (using power regression) was observed between SCL and body mass in all tortoises, including hatchlings, juveniles and adults (*R^2^* = 0.97, *P* < 0.01, *N* = 90). Fisher’s exact tests revealed that adult tortoises were significantly more likely to have intraerythrocytic hemogregarine gametocytes (*P* = 0.001) and more likely to have ticks (*P* = 0.002) than juvenile tortoises. There were no significant differences between age classes for PCR results for *Ranavirus*, *Herpesvirus* or *Anaplasma* spp., or for polychromasia, anisocytosis or intraerythrocytic inclusions suggestive of *Anaplasma* spp. infection (all *P* > 0.05).

Fisher’s exact tests showed that adult tortoises were significantly more likely to have clinical signs of URTD compared to juveniles (*P* = 0.002). Tortoise sex was not significantly related to *Mycoplasma* spp. serology results (all *P* > 0.05). Logistic regression models (Table 5) revealed statistically significant, positive relationships between the presence of antibodies to *Mycoplasma* spp. and SCL, PCV and plasma concentrations of albumin, alpha-2 globulin, beta globulin, and gamma globulin. Total WBC estimates and heterophil and lymphocyte counts were not significantly related to *Mycoplasma* spp. serology results (all *P* > 0.05).

**Table 5:** Results of logistic regression analysis to examine the relationships of *Mycoplasma* spp. serology results with SCL, hematology parameters and plasma protein concentrations for both sampling sites combined

	Data for explanatory variables					Model fit		
Variable	Coefficient	SE	*P*	Odds ratio	95% CI	χ^2^	*P*	df
*Mycoplasma* spp. antibodies	--	--	--	--	--	--	--	--
SCL	0.03	0.01	<0.001^*^	1.03	1.02–1.05	39.96	<0.001	1
PCV	0.19	0.07	0.010^*^	1.20	1.05–1.39	8.23	0.004	1
Estimated tWBC	0.03	0.07	0.697	1.03	0.90–1.18	0.15	0.695	1
Mature heterophils	0.08	0.17	0.642	1.08	0.77–1.52	0.22	0.640	1
Immature heterophils	1.03	1.41	0.465	2.79	0.18–43.89	0.56	0.454	1
Lymphocytes	−0.03	0.19	0.878	0.97	0.67–1.41	0.02	0.878	1
Pre-albumin	2.12	1.95	0.277	8.30	0.183–377.08	1.26	0.262	1
Albumin	5.19	2.21	0.019^*^	179.35	2.37–13559.08	6.81	0.009	1
Alpha-1 globulins	13.14	7.10	0.064	8908.89	0.46–6515.69	3.85	0.050	1
Alpha-2 globulins	11.21	3.14	<0.001^*^	73956.78	156.53–42153.61	20.79	<0.001	1
Beta globulins	2.46	0.70	<0.001^*^	11.74	2.99–46.17	22.00	<0.001	1
Gamma globulins	11.76	3.19	<0.001^*^	28424.73	247.65–98824.29	28.79	<0.001	1

Abbreviations: SE, standard error.

^*^Statistically significant associations.

Tortoises with ticks were significantly more likely to have intraerythrocytic hemogregarine gametocytes noted on examination of blood films (*P* < 0.001). Logistic regression showed a significant association between PCV and presence of intraerythrocytic inclusions suggestive of *Anaplasma* spp., but not between PCV and qPCR assay results (Table 6). All of the tortoises with intraerythrocytic inclusions suggestive of *Anaplasma* spp. also had polychromasia, and all but one also had mild to moderate anisocytosis. Cohen’s Kappa coefficient indicated a slight level of diagnostic agreement between *Anaplasma*-like inclusions viewed during microscopic evaluation of blood films and the results of qPCR assays targeting *Anaplasma* spp. DNA (κ = 0.14, SE of κ = 0.06, 95% CI = 0.02–0.26). Fisher’s exact tests showed no statistically significant relationships between presence of *A. tuberculanum* ticks and *Anaplasma* spp. infection (diagnosed via either identification of intraerythrocytic inclusions suggestive of *Anaplasma* spp., or via qPCR), or between *Anaplasma* spp. infection and *Mycoplasma* spp. serology results (all were *P* > 0.05).

**Table 6:** Results of logistic regression analysis to examine the relationships between PCV and *Anaplasma* spp. diagnostics, including presence/absence of intraerythrocytic inclusions and qPCR results, for both sampling sites combined. ^*^Statistically significant associations

	Data for explanatory variables					Model fit		
Variable	Coefficient	SE	*P*	Odds ratio	95% CI	χ^2^	*P*	df
Intraerythrocytic inclusions suggestive of *Anaplasma* spp. vs. PCV	−0.17	0.09	0.048^*^	0.84	0.71–1.00	4.35	0.037	1
*Anaplasma* spp. qPCR results vs. PCV	0.07	0.06	0.230	1.07	0.96–1.20	1.48	0.224	1

## Discussion

### Hematology and plasma biochemistry parameters

The hematology data presented here fall within previously determined reference intervals for gopher tortoises (Taylor and Jacobson, 1982, Rosenberg *et al*., 2018) and were not indicative of active clinical disease. The plasma protein electrophoretic profiles were generally higher than those reported by Rosenberg *et al*. (2018) for a healthy captive group of gopher tortoises. Because consistent methodologies were used for both studies, these differences are likely real and may indicate a higher degree of antigenic stimulation in the wild gopher tortoises sampled in this study. This is consistent with the fact that free-ranging animals typically have higher internal and external parasite burdens and are likely exposed to pathogens more frequently than captive animals, which are often regularly treated with parasiticides and also receive supportive care, including anti-microbials, when sick (Jacobson *et al*., 1998). There were four juvenile tortoises in this study with PCV values less than 20% (range: 15%–19%). Two of these samples were collected from the brachial vein and two were collected from the jugular vein. While PCV values ranging from 14% to 34% are considered ‘normal’ for healthy gopher tortoises (Hernandez *et al*., 2011, Rosenberg *et al*., 2018, this study), PCV values less than 20% are on the low end of the reference intervals calculated for tortoises in this study. There were no data, however, to suggest that these tortoises were unhealthy.

The presence of higher numbers of immature heterophils in adult tortoises indicates active inflammation, which along with the increased concentrations of plasma proteins suggests antigenic stimulation (Stacy *et al*., 2011, Zaias and Cray, 2002). The progression toward increased plasma proteins as animals mature, especially with regards to beta globulin and gamma globulin (Fig. 4**)**, reflects the increased length of time that adult tortoises have been exposed to various parasites and pathogens compared to juveniles, and to mount immune responses to them (Zimmerman *et al*., 2013). Increased antigenic stimulation in older tortoises may also be related to increased movement and social interactions during mating season, as adult males increase movement to visit females and thus promote exposure to pathogens, or to hormone production, as vitellogenin in reproductive females is associated with increased globulins (Campbell, 2006; Berish and Medica, 2014). The observed correlations between PCV and plasma hemoglobin concentration and between TP-R and TP-B (Fig. 3) show that, in circumstances wherein it may be logistically difficult to measure hemoglobin and/or total protein, PCV and TP-R may be used as suitable proxies to estimate hemoglobin and total protein in plasma without any visible discoloration (i.e. hemolysis), respectively (Rosenberg *et al*., 2018, Stacy *et al*., 2019, Fleming *et al*., 2020). Interestingly, hemoglobin concentration using the SI unit g l^−1^ was found to be about three times the PCV, similar to mammals and other non-mammalian species (Stacy *et al*., 2019).

### Infectious disease prevalence estimates

Overall, 42.9% of all tortoises tested had circulating antibodies to *M. agassizii*. At both study sites, adult tortoises were significantly more likely to have clinical signs of URTD than juveniles and there was a significant relationship between tortoise size (SCL) and *M. agassizii* antibody test results. Previous studies have shown a correlation between carapace length and antibody prevalence, with juvenile turtles less likely to have antibodies to *Mycoplasma* spp. (Beyer, 1993, Wendland, 2007) except in populations undergoing epizootic events (Wendland, 2010). Interestingly, in this study we detected antibodies to *M. agassizii* in 8 juveniles sampled at Loggerhead Park, representing 29% of the juveniles sampled at that site. This result, along with the detection of antibodies to *M. agassizii* in 70% of the adults sampled at Loggerhead Park, suggests that this tortoise aggregation may be undergoing an *M. agassizii* epizootic. This finding may be related to density-dependent factors, since increased gopher tortoise population density has been documented to result in factors increasing opportunities for social interactions, including higher incidences of shared burrows, greater home range overlap and increased mating attempts (Guyer *et al*., 2012). In contrast, although the total prevalence of tortoises with antibodies to *M. agassizii* was higher at HBOI, only 1 juvenile tortoise (14%) sampled at HBOI had antibodies to *M. agassizii.* No tortoises at either sampling site were confirmed to have antibodies to *M. testudineum*, although 11% of tortoises sampled at Loggerhead Park had ‘suspect positive’ titers. Antibodies to *M. testudineum* in gopher tortoises have typically been shown in tortoises captured in more northern latitudes, including the northeastern parts of FL (Wendland, 2007, Diemer-Barish *et al*., 2010) and in multiple sites across GA, USA (McGuire *et al*., 2014a). Despite the relatively high prevalence of antibodies to *M. agassizii* at both sites (51% at Loggerhead Park and 63% at HBOI), the prevalence of clinical signs associated with past and current URTD was lower (14% and 29%, respectively). This, along with the lack of a significant correlation between presence of antibodies to *Mycoplasma* spp. and clinical signs of URTD, may be explained by the fact that detection of circulating antibodies is typically associated with past or chronic infection (Jacobson *et al*., 1995), while clinical signs of URTD may signify current mycoplasmal infections in tortoises that have not had time to seroconvert (Diemer-Barish *et al*., 2010). In these instances, infection with another mycoplasmal species or other respiratory pathogens must also be considered (Wendland, 2007). The presence of a statistically significant relationship between antibodies to *M. agassizii* and PCV and plasma alpha-1 globulin, beta globulin and gamma globulin concentrations is intriguing, particularly since the beta and gamma protein fractions contain antibodies. Both PCV and total globulins were also positively correlated to tortoise size; therefore, size and/or hydration status may be a confounding variable in these results. While the effects of mycoplasmosis on the long-term health and viability of gopher tortoise populations is not well understood, it seems likely that physiological stress associated with extrinsic stressors including human impacts on tortoises and their habitats and population density are related to both overt morbidity and mortality, as well as seroconversion that is detectable via molecular assays (Jacobson *et al*., 2014).

None of the tortoises tested positive for *Ranavirus* or *Herpesvirus* via PCR; this represents important baseline data, since these viruses are thought to be emerging pathogens of other tortoise and turtle species (Johnson *et al*., 2005, 2008, 2010, Jacobson *et al*., 2012). Adult tortoises were significantly more likely than juvenile tortoises to have both ticks and intraerythrocytic hemogregarine gametocytes, and tortoises with ticks were significantly more likely to have intraerythrocytic hemogregarine gametocytes. These results are noteworthy because hemogregarines in tortoises are thought to be transmitted by ticks (Cook *et al*., 2009). Differences observed between sampling sites also support this hypothesis, since ticks were found on 59% and hemoparasites were identified in 30% of the tortoises sampled at HBOI, while only a single tick was found, and no hemoparasites were identified in tortoises sampled at Loggerhead Park. These hemoprotozoans were considered an incidental finding in these cases (Stacy *et al*., 2017); in general, the clinical significance of hemoparasite infections is related to the level of infection and other stressors (Hernandez *et al*., 2011). The hemoprotozoans were not identified to the species level, since it is not possible to speciate them based on morphological characteristics alone, and molecular characterization was not performed in this study (Hernandez *et al*., 2011).

Upon hematological examination, variably-sized (2–5 μm), round to oval, basophilic, stippled, intracytoplasmic inclusions were observed within erythrocytes of 7 tortoises (14%) captured at Loggerhead Park, and in 1 (3%) tortoise captured at HBOI. These inclusions were consistent with previous descriptions of *Anaplasma* spp., a bacterial hemoparasite associated with anemia and an emerging pathogen in gopher tortoises (Crosby *et al*., 2016, Wellehan *et al*., 2016, Raskin *et al*., 2020). Although *Anaplasma* infections in other species are known to be transmitted by ticks (Vanstreels *et al*., 2018), in this study statistically significant relationships were not found between the presence of *A. tuberculanum* ticks and intraerythrocytic inclusions, nor between ticks and blood samples that were positive for *Anaplasma* spp. via qPCR. Ticks were not tested for *Anaplasma* spp. using qPCR in this study; future studies should include directly testing ticks for this emerging pathogen. Anaplasmosis in previously reported gopher tortoise cases has been associated with anemia that was attributed to hemolytic disease (Raskin *et al*., 2020). Here, statistical analysis revealed a significant, negative association between PCV and presence of intraerythrocytic inclusions suggestive of *Anaplasma* spp. Additionally, all tortoises with *Anaplasma*-like inclusions also had polychromasia, an indication of increased release of erythrocytes from hematopoietic tissues, and all but one also had anisocytosis, characterized by erythrocytes of unequal size. None of these tortoises had abnormally colored plasma samples that would be indicative of hemolysis. Both polychromasia and anisocytosis can be associated with regenerative anemia in reptiles; thus, this observation could indicate underlying hemolysis and continued erythrocyte regeneration in *Anaplasma* spp.-infected tortoises (Jackson, 2007, Stacy *et al*., 2011). The mean ± SD PCV for tortoises with *Anaplasma-*like intraerythrocytic inclusions was 23 ± 7, while the mean ± SD PCV for tortoises without these inclusions was 28 ± 5; however, only one of the tortoises with *Anaplasma-*like intraerythrocytic inclusions was clinically anemic based on previously published reference intervals (Rosenberg *et al*., 2018). Moreover, lymph dilution of the blood sample from this tortoise cannot be excluded since TP-R and TP-B were also low (<20.0 g L^−1^ and 10.0 g L^−1^, respectively), and the tortoise was not underweight and did not appear clinically ill. There was no statistically significant relationship between PCV and qPCR assay results. Interestingly, the Loggerhead Park tortoise aggregation with higher frequency of *Anaplasma* spp. had heterophil projections, which were absent in tortoises at HBOI. These heterophil projections are considered artifact or associated with inflammation (non-specific) (Stacy *et al*., 2017). Given consistent sample handling and processing times at both sites, artifact is less likely. An association with the presence of a pathogen such as *Anaplasma* spp., or other pathogens, and an immune response is plausible. There was only a slight level of diagnostic agreement between *Anaplasma*-like inclusions viewed during microscopic evaluation of blood films and the results of qPCR assays targeting *Anaplasma* spp. DNA. This result was driven by the number of negative agreements between the two diagnostic techniques (*N* = 49). While PCR is likely a more sensitive method for diagnosing this blood parasite, infection confirmation is most reliable when the two diagnostic methods are applied in tandem, since the number of organisms may be too low for PCR detection or be missed by blood film review, respectively. Because there were no tortoises that had both the inclusions and a positive qPCR result, it is difficult to make conclusive statements about the presence and significance of Anaplasmosis in these gopher tortoise aggregations.

## Conclusions

This work contributes important baseline health information on gopher tortoises toward the southern end of the species’ range. Because the gopher tortoise is one of the most commonly translocated species in North America (Tuberville *et al*., 2011; Cozad *et al*., 2020), it is important to understand pathogen distributions within their populations (McGuire *et al*., 2014b). This study highlights the importance of continued health surveillance of gopher tortoise populations, as we detected an emerging pathogen (*Anaplasma* spp.), documented the absence of two other emerging pathogens (*Herpesvirus*, *Ranavirus*) and provided evidence for a potential *M. agassizii* epizootic within the tortoise aggregation inhabiting Loggerhead Park. Long-term studies of these and other populations of management concern will help us to better understand the consequences of disease and various stressors on important variables including behavior and reproductive potential (McGuire *et al*., 2014a, 2014b). Thus, further health assessments and pathogen surveillance in the gopher tortoises of southeastern FL are warranted.

## Funding

This work was supported by grants from Association of Reptile and Amphibian Veterinarians, Chicago Herpetological Society, Wildlife Disease Association Challenge in association with experiment.com; generous donations from the Albert E. and Birdie W. Einstein Fund, Bonnie Simes; and various donors to our crowdfunding efforts to fund this project. Summer internship funds were provided by the Link Foundation [to K.R.] and by the James Pomponi Memorial Scholarship Fund [to C.X.].
